# Meetings of Societies

**Published:** 1946

**Authors:** 


### Meetings of Societies

### Bristol Medico-Chirurgical Society

MEETINGS OF THE PRESENT SESSION, 1945–46.

THE first meeting of the season was held in October, when Dr. H. H. Carleton, D.M., F.R.C.P., gave his Presidential Address entitled "**Nature and Science,**" which was received with great interest.

At this Meeting the Officers for the succeeding year were elected.

*President*, Dr. H. H. Carleton.
*President-elect*, Mr. Hubert Chitty.
*Hon. Secretary*, Mr. A. L. Eyre-Brook.
*Hon Treasurer*, Dr. G. E. F. Sutton.

*Honorary Medical Librarians.* It was decided to ask the Professors of Medicine, Surgery, Gynæcology and Midwifery and Pathology to act as Honorary Medical Librarians.

---

The Second Meeting was held in November, when Mr. Norman Tanner, F.R.C.S., opened a discussion on the "**Management of Chronic Gastric and Duodenal Ulcer.**" This paper was extremely well received and stimulated a very interesting and prolonged discussion on this difficult problem.

The following were elected to the Committee: Dr. S. Bryan Adams, Dr. R. F. Barbour, Dr. Frank Bodman, Dr. Charles Corfield, Dr. Beryl Corner, Dr. G. L. Ormerod, Professor T. Hewer, Dr. G. D. Kersley, Dr. K. Soltau.

---

A Clinical Meeting followed in January at the Bristol Royal Infirmary, Out-Patients' Department. About eighty cases of interest were demonstrated, and the success of the evening was in no small part due to the excellent refreshments provided by the Matron and Nursing Staff, and the practical assistance in all the arrangements provided by Mr. Hill and his staff.

---

The Fourth Meeting was held in February, when two papers on "**The Diagnosis of Tumours of the Central Nervous System,**" were given by Miss Diana Kinlock Beck, F.R.C.S., and Professor Hugh Cairns, F.R.C.S., the latter illustrating his paper with an excellent series of slides.

### BRISTOL MEDICO-CHIRURGICAL SOCIETY.

**INCOME AND EXPENDITURE ACCOUNT for the seven years ended 30th September, 1945.**

| EXPENDITURE. | £ | s. | d. | £ | s. | d. |
|---|---|---|---|---|---|---|
| Grants to *The Bristol Medico-Chirurgical Journal* .. .. .. .. | | | | 760 | 0 | 0 |
| Grants to Medical War Relief Fund | | | | 348 | 10 | 0 |
| Librarian—Honorarium .. .. .. .. | | | | 105 | 0 | 0 |
| Miscellaneous Expenses— | | | | | | |
| Catering .. .. .. .. | 30 | 18 | 2 | | | |
| Postages and Sundries | 61 | 0 | 8 | | | |
| Printing and Stationery | 29 | 8 | 10 | | | |
| Audit Fee (7 years) .. | 14 | 14 | 0 | | | |
| Lantern and Cinematograph at Meetings .. | 2 | 6 | 0 | | | |
| Lewis's Medical and Scientific Library—Subscription and Postages .. .. .. .. .. | 45 | 4 | 3 | | | |
| | | | | 183 | 11 | 11 |
| | | | | 1,397 | 1 | 11 |
| Surplus for the 7 years .. .. .. | | | | 46 | 18 | 5 |
| | | | | £1,444 | 0 | 4 |

| INCOME. | £ | s. | d. |
|---|---|---|---|
| Members' Subscriptions .. .. .. .. | 1,354 | 12 | 6 |
| Interest on Bank Deposits .. .. | 13 | 2 | 10 |
| Interest on £500 4 per cent. Consolidated Stock, less tax .. .. | 76 | 5 | 0 |
| | £1,444 | 0 | 4 |

**BALANCE SHEET as at 30th September, 1945.**

| LIABILITIES AND CAPITAL. | £ | s. | d. | £ | s. | d. |
|---|---|---|---|---|---|---|
| Sundry Creditors .. .. | | | | 14 | 14 | 0 |
| Capital as at 30th Sept., 1938 .. .. .. .. | 1,134 | 6 | 9 | | | |
| *Add* Surplus for the 7 years .. .. .. | 46 | 18 | 5 | | | |
| | | | | 1,181 | 5 | 2 |
| | | | | £1,195 | 19 | 2 |

| ASSETS. | £ | s. | d. | £ | s. | d. |
|---|---|---|---|---|---|---|
| Cash at Bank— | | | | | | |
| Current Account .. .. | 569 | 2 | 10 | | | |
| Deposit Account .. .. | 189 | 7 | 4 | | | |
| | | | | 758 | 10 | 2 |
| £500 4 per cent. Consolidated Stock at cost .. .. .. .. .. .. | | | | 437 | 9 | 0 |
| | | | | £1,195 | 19 | 2 |

Audited and found correct,

H. E. KEELER,

*Chartered Accountant,*

*Auditor.*

16–18 Clare Street, Bristol 1.

14*th May*, 1946.

The retiring Secretary, Mr. R. V. Cooke, F.R.C.S., was unable to attend the Fourth Meeting because of illness. The President expressed on behalf of the Society their profound thanks to Mr. Cooke for his untiring service over a great number of years.

---

The Fifth Meeting took place in March, when Dr. G. D. Kersley, F.R.C.P., gave a stimulating address on "**The Diagnosis and Treatment of Rheumatoid Arthritis,**" which was followed by a discussion in which many took part.

So far thirty-four new members have been elected, and it has been decided that temporary Honorary Membership is automatically conferred on all ex-members of the Forces while they are engaged on post-graduate work in Bristol.

The sum of £250 has been voted by the Society to the Medical War Relief Fund.

